# The Involvement of Peripheral and Brain Insulin Resistance in Late Onset Alzheimer’s Dementia

**DOI:** 10.3389/fnagi.2019.00236

**Published:** 2019-09-06

**Authors:** Jaume Folch, Jordi Olloquequi, Miren Ettcheto, Oriol Busquets, Elena Sánchez-López, Amanda Cano, Triana Espinosa-Jiménez, Maria Luisa García, Carlos Beas-Zarate, Gemma Casadesús, Mónica Bulló, Carme Auladell, Antoni Camins

**Affiliations:** ^1^Department of Biochemistry and Biotechnology, Faculty of Medicine and Health Sciences, University Rovira i Virgili (URV), Reus, Spain; ^2^Berlin Institute of Health (BIH), Zoologisches Institut, Technische Universität Braunschweig, Braunschweig, Germany; ^3^Biomedical Research Networking Centre in Neurodegenerative Diseases (CIBERNED), Institute of Health Carlos III, Madrid, Spain; ^4^Instituto de Ciencias Biomédicas, Facultad de Ciencias de la Salud, Universidad Autónoma de Chile, Talca, Chile; ^5^Departament de Farmacologia, Toxicologia i Química Terapèutica, Facultat de Farmàcia i Ciències de l’Alimentació, Universitat de Barcelona, Barcelona, Spain; ^6^Institut de Neurociències, Universitat de Barcelona, Barcelona, Spain; ^7^Unitat de Farmàcia, Tecnologia Farmacèutica i Fisico-Química, Facultat de Farmàcia i Ciències de l’Alimentació, Universitat de Barcelona, Barcelona, Spain; ^8^Institute of Nanoscience and Nanotechnology (IN2UB), University of Barcelona, Barcelona, Spain; ^9^Laboratorio de Regeneración y Desarrollo Neural, Departamento de Biología Celular y Molecular, Instituto de Neurobiología, CUCBA, Guadalajar, México; ^10^Department of Biological Sciences, Kent State University, Kent, OH, United States; ^11^Centro de Investigación Biomédica en Red Fisiopatología de la Obesidad y la Nutrición (CIBEROBN), Institute of Health Carlos III, Madrid, Spain

**Keywords:** insulin resistance, obesity, type 2 diabetes mellitus, Alzheimer’s disease, Mediterranean diet, neuroinflammation and neurodegeneration

## Abstract

Nowadays, Alzheimer’s disease (AD) is a severe sociological and clinical problem. Since it was first described, there has been a constant increase in its incidence and, for now, there are no effective treatments since current approved medications have only shown short-term symptomatic benefits. Therefore, it is imperative to increase efforts in the search for molecules and non-pharmacological strategies that are capable of slowing or stopping the progress of the disease and, ideally, to reverse it. The amyloid cascade hypothesis based on the fundamental role of amyloid has been the central hypothesis in the last 30 years. However, since amyloid-directed treatments have shown no relevant beneficial results other theories have been postulated to explain the origin of the pathology. The brain is a highly metabolically active energy-consuming tissue in the human body. It has an almost complete dependence on the metabolism of glucose and uses most of its energy for synaptic transmission. Thus, alterations on the utilization or availability of glucose may be cause for the appearance of neurodegenerative pathologies like AD. In this review article, the hypothesis known as Type 3 Diabetes (T3D) will be evaluated by summarizing some of the data that has been reported in recent years. According to published research, the adherence over time to low saturated fatty acids diets in the context of the Mediterranean diet would reduce the inflammatory levels in brain, with a decrease in the pro-inflammatory glial activation and mitochondrial oxidative stress. In this situation, the insulin receptor pathway would be able to fine tune the mitochondrial biogenesis in neuronal cells, regulation the adenosine triphosphate/adenosine diphosphate intracellular balance, and becoming a key factor involved in the preservation of the synaptic connexions and neuronal plasticity. In addition, new targets and strategies for the treatment of AD will be considered in this review for their potential as new pharmacological or non-pharmacological approaches.

## Introduction

Several theories have been in the headlines of the Alzheimer’s disease (AD) research scene in the last 10 years (Folch et al., 2013; Alzheimer’s Association, 2016; Dobson, 2017; Hurtado-Puerto et al., 2018) and, in many of them, it was postulated how an alteration in a metabolic mechanism runs in parallel, or is the cause, for the development of the classical features of AD (Frölich et al., 2015). Eventually, it has led to the understanding that pathologies like type 2 Diabetes Mellitus (T2DM) and AD, or conditions like morbid obesity, previously believed to run independently, are actually highly connected through specific molecular interactions that evolve in the same direction (de la Monte, 2012; De Felice et al., 2014).

Despite the hypotheses focus on amyloid and TAU phosphorylation have dominated the neuropathology of AD so far, new theories have come to light, in part, due to the failure of all the developed drugs directed against molecules related to the amyloidogenic pathway (Gauthier et al., 2016). It has been described that this failure may be the result of the complex etiology of AD. As of now, it could be classified into two subgroups. On the one hand, familial AD in which genetic alterations of the amyloid precursor protein (APP) or presenilin-1 (PS-1) are involved, accounting the 3% of cases of the disease. On the other hand, representing the remaining 97%, the late onset form of AD (LOAD) which is associated with advanced age, mutations of the apolipoprotein E (APOE) ε4 allele, hypertension and hyperlipidaemia, as well as with coronary disease, T2DM and obesity among other less determinant factors.

In parallel with AD, obesity has become pandemic in the Western world and it has shown increased prevalence in most countries mainly due to changes in nutritional habits and lifestyle (de la Monte, 2014; Luciano et al., 2015; Kivipelto et al., 2018). Although the interaction between obesity, aging and AD is a complex process, there are evidences demonstrating that obesity and metabolic dysfunctions in middle age substantially increase the risk of developing AD (Kivipelto et al., 2018). Thus, studies conducted in the last 20 years support the hypothesis that deficits in brain insulin and insulin-like growth factor (IGF-1) signaling mediate cognitive impairment and neurodegeneration in AD (de la Monte, 2012). As we will discuss below, the main role of insulin and insulin receptor pathway in different brain regions as hippocampus and cortex is mainly related to the maintenance of intracellular energy levels needed to sustain synaptogenesis and neuronal plasticity. For this reason, some researchers have even referred to AD as Type 3 Diabetes (T3D; de la Monte, 2012, 2014; Chami et al., 2016; Kandimalla et al., 2017; Kang et al., 2017; Tong et al., 2017). This concept describes a brain-specific pathological situation in which insulin and IGF resistance is developed inducing cognitive impairments and neurodegeneration. Insulin resistance is classically defined as the state in which high levels of circulating insulin (hyperinsulinemia) are associated to hyperglycemia, concept that has been extended to other tissues and organs which show reduced activation of the pathways in insulin signaling. Therefore, AD and diabetic pathologies share several common features (Willette et al., 2015), situation in which adherence to good life habits, especially in terms of motor activity and diet, may have beneficial effects on cognitive processes during aging in humans.

From a metabolic outlook, the brain requires a high amount of energy to maintain its functions, especially for the transmission of synaptic impulses (Yun and Hoyer, 2000; Hoyer, 2004; Maurer and Hoyer, 2006). Hence, alterations in brain glucose metabolism lead to severe dysregulations in cellular function. For example, a decrease in ATP production and choline acetyltransferase activity in presynaptic cholinergic neurons reduce the availability of acetylcholine in the brain which is worsened by the depletion of citric acid cycle intermediates that are also required for the synthesis of this neurotransmitter (Hoyer, 2002a,b; Salkovic-Petrisic et al., 2009). Alterations on its availability could also trigger a dysfunction in synaptic transmission and affect proper cognitive function (Hoyer, 2004; Frölich et al., 2015; Fadel and Reagan, 2016).

This review article focuses on the evidence concerning the effects of obesity and T2DM in the process of cognitive loss. Likewise, we discuss throughout the manuscript the close relationship between alterations at the peripheral level related to obesity that may favor the risk of developing AD and the role of amyloid-β (Aβ) generated at the brain level, which could be an inducer of the onset of T2DM. Since obesity and metabolic syndrome are modifiable risk factors that contribute to insulin-resistant diabetes, special emphasis will be put on their relationship with T3D. Finally, pharmacological and environmental interventions, such a suitable diet, will also be discussed as possible strategies to prevent obesity-related cognitive loss.

## Physiological Role of Insulin Beyond Peripheral Tissues

As we mentioned, the classical amyloid and Tau hypotheses of AD have been challenged by reported evidence that describes how they may not be the source for the pathology in its late onset form (Selkoe and Hardy, 2016). It was Dr. Hoyer who in 1985 introduced the idea of alterations in brain metabolism as the source for the appearance of LOAD through defects in the oxidative degradation of glucose (Hoyer, 2004; Frölich et al., 2015; Morgen and Frölich, 2015). Specifically, it has been described that oxidative dysregulation triggers cellular downstream signaling pathways promoting neuronal degeneration through the induction of stress in organelles like the mitochondria and the endoplasmic reticulum. As a consequence, Aβ and Tau alterations would emerge contributing to the ongoing cognitive loss. This could explain why drugs focusing on Aβ and Tau have proven useless so far since they would be targeting a consequence or aggravator of the pathology but not its root.

In addition to this, in recent years it has been demonstrated that insulin receptors (IRs) and their ligand hormone play a more relevant role in the brain than was previously thought. Specifically, several studies have reported presence of IRs in hippocampus, cerebral cortex, cerebellum and choroidal plexus of the mammalian brain (Hoyer, 2004; de la Monte et al., 2006) and especially in postsynaptic terminals (Abbott et al., 1999). It has been demonstrated that insulin levels become strongly reduced in these regions during aging and in sporadic AD (Ramalingam and Kim, 2014). Indeed, insulin is preferentially transported to the brain through the blood-brain barrier (BBB) in a receptor-mediated mechanism and its availability is critical for the activation of brain IR. In this aspect, it has been proposed that brain insulin has a neuroprotective capacity against the accumulation of senile plaques, by regulating Aβ peptide levels and preventing the binding of Aβ oligomers to synapses (Zhao et al., 2008; De Felice et al., 2009, 2014; Lyra E Silva et al., 2019). Interestingly, AD preclinical models show that preventing IR inhibition enhances Protein kinase B (AKT) signaling, which is involved in cell proliferation, cell growth, protein synthesis and inhibition of apoptosis, but also in the hyperphosphorylation of Tau through the control of the glycogen synthase kinase 3β (GSK3β; El Khoury et al., 2014). By contrast, insulin deficits have been linked to the inhibition of several phosphatases involved in Tau dephosphorylation (El Khoury et al., 2014). Finally, it has been proven that insulin signaling pathway is also involved in the modulation of neuroinflammatory processes and vascular inflammation (Chen and Zhong, 2013). Likewise, insulin modulation acts through the mitogen-activated protein kinase (MAPK) pathway, which plays an important role in cell differentiation, cell proliferation, apoptosis as well as inflammation. As we will discuss below, the activation of some c-JUN N-terminal Kinases (JNKs) should be a key factor linking insulin signaling to synaptogenesis failure (Huang et al., 2017; Pomytkin et al., 2018).

In 2000, Bru and co-workers published an interesting article demonstrating that brain IR is involved in the metabolic control of peripheral tissues through the generation of a murine model with a specific deletion of the neuronal IR gene (NIRKO mice; Bru et al., 2000). This inactivation resulted in insulin resistance at the central nervous system (CNS) but mice also developed obesity, combined with hyperphagia, an increase of leptin and insulin concentrations in plasma, as well as the development of hypertriglyceridemia. In agreement with this, Obici and co-workers demonstrated that a decrease in the hypothalamic IR expression is enough to induce several key features of metabolic syndrome (Obici, 2009). Hence, insulin resistance in the brain would be involved in the pathophysiology of obesity and T2DM in the peripheral tissues.

All these experimental data indicate that brain IR plays a key role in: (i) regulation of cognitive processes through hippocampal IR; and (ii) regulation of peripheral glucose metabolism. Consequently, restoration of normal insulin levels and prevention of brain insulin resistance may be a therapeutic strategy for delaying cognitive loss in AD (Biessels, 2013; Ramalingam and Kim, 2014).

## Towards Brain Insulin Resistance in Late Onset Alzheimer’s Disease

Recently, Castellani et al. (2019) proposed, in an excellent review on AD therapeutics, that it was very reasonable to conclude that the amyloidogenic pathway is very closely related to AD, however, the important role of other players was suggested into the origin of the pathology since the clearance of Aβ protein was not enough to modify the evolution of the disease. Furthermore, pharmacological strategies targeting Aβ-related biomarkers like BACE1 inhibitors or anti-Aβ antibodies, have failed to cure or halt LOAD (Cummings et al., 2018). Hence, new strategies should be designed to complement the actual therapeutic proposals that, so far, have been mainly focused on the amyloid hypothesis.

One of the first reviews that criticized the paradigm of the amyloid cascade overactivation as the origin of AD was written by one of the co-authors that first described this central hypothesis (Cleary et al., 2005; Hardy, 2009; Selkoe and Hardy, 2016). But, back in 1985, as it was previously mentioned, before the installment of the amyloidogenic hypothesis, Dr. Siegfried Hoyer had already proposed the concept of central insulin resistance and altered insulin signaling in LOAD (Frölich et al., 2015; Hoyer, 2002b, 2004; Morgen and Frölich, 2015). Later on, Dr. de la Monte published a series of articles about the metabolic hypothesis of AD, and became one of the main defenders of the so-called “brain insulin resistance” or T3D hypothesis (de la Monte et al., 2006; de la Monte and Wands, 2008; Chami et al., 2016; de la Monte, 2017). In these experiments, the intracerebral administration of streptozotocin (STZ) was used as an experimental model to induce LOAD-like cognitive impairments in rodents, allowing to study this theory deeper (de la Monte et al., 2006; Correia et al., 2011; Salkovic-Petrisic et al., 2013; Tong et al., 2017). The research by Dr. de la Monte in AD patients also proved significant reductions in insulin and IGF-1 receptor levels in the frontal cortex, hippocampus, and hypothalamus (Steen et al., 2005), reinforcing the idea of T3D. Likewise, Cardoso et al. (2017) suggested the term “diabesity” to explain the presence of both metabolic and cognitive affectations.

In parallel with these observations, results from epidemiologic studies contributed to reinforce the proposed concept of T3D. Doubtlessly, one of the most important is the Rotterdam’s study (Ott et al., 1999; Schrijvers et al., 2010; Luciano et al., 2015). This project has investigated the connection between T2DM and LOAD for almost a decade, revealing that those patients diagnosed with T2DM had higher risk of developing dementia. Subsequent clinical and epidemiological studies have confirmed this association, by demonstrating that alterations of metabolic parameters related to glucose metabolism were associated with cognitive loss (Talbot et al., 2012; Biessels, 2013; Walker and Harrison, 2015). Moreover, in a clinical study in patients with AD and hyperinsulinemia, Willette et al. (2015) demonstrated that insulin resistance increased the number of Aβ depositions in the brain. It has also been reported that neurons from AD patients exhibit an insulin mRNA expression four times lower than normal in the hippocampus and two times lower than normal in the hypothalamus (Chen and Zhong, 2013). This situation would be aggravated by the disruption of the activity of astrocytes forming the BBB. Likewise, patients with AD show alterations in the transport of this hormone into the brain (Molofsky et al., 2012; An et al., 2018). It is noteworthy to point out that, in addition to the lower availability of insulin in the brain, cerebral insulin resistance is another pathophysiological feature of AD. Thus, it seems that insulin signaling could have a key role in cognitive loss.

In 2014, the Hisayama’s study showed, through the analysis of microarrays, modifications in the normal expression of genes related to T2DM in AD brains, especially in the hippocampus (Hokama et al., 2014). More recent studies have also reinforced the notion of an insulin resistance having a key role in AD’s pathogenesis. For instance, An et al. (2018) reported a strong association between impairments in glucose metabolism and increased glucose concentrations in areas sensible to Aβ deposition and neurofibrillary pathology. The authors suggested that AD is associated with a failure in neuronal glucose utilization, which is mediated by an alteration in glycolysis. The authors argued that three enzymes involved in the glycolysis (hexokinase, phosphofructokinase, and pyruvate kinase) showed significant reduction in their activity in AD. In turn, in the Whitehall II clinical study, Singh-Manoux et al. (2018) reported that obesity (BMI >30 kg/m^2^) at 50 years of age is a risk factor for AD. However, the association decreased with increasing age, indicating that this association is modified by age and obesity, being midlife obesity the riskiest stage for dementia (Singh-Manoux et al., 2018). In another interesting clinical study, Ahmed et al. (2017) reported a bidirectional association between T2DM and LOAD. Besides, they reported that Memantine—a drug which is currently used in AD treatment- showed an ameliorating effect on T2DM. This is in accordance with results published by our research group and others, where the benefits of Memantine administration were demonstrated in a mixed murine model of T2DM and AD (Sato and Morishita, 2013; Shinohara and Sato, 2017; Ettcheto et al., 2018b; Deng et al., 2019).

Regarding other preclinical studies, have been shown that hyperglycemia raises Aβ levels in the interstitial fluid (ISF) by altering neuronal activity. It seems that high glucose metabolism can alter ATP-sensitive potassium (KATP) channels, which are the link between changes in metabolism, neuronal activity and ISF Aβ (Macauley et al., 2015; Stanley et al., 2016). In turn, Grillo et al. (2015) reported that the administration of viral vectors expressing an antisense sequence of the rat brain IR caused cognitive impairments. Thus, they generated a specific rat model of altered brain insulin signaling associated to cognitive loss. These results are of great relevance since demonstrated that selective insulin resistance at the hippocampal level contributes directly to the development of cognitive deficits observed in patients with metabolic disorders such as T2DM and obesity (Fadel and Reagan, 2016).

Finally, among other risk factors likely to favor the development of sporadic AD, the mutation of the APOE4 allele has recently shown an intriguing association with insulin resistance. Thus, it has been suggested that APOE4 impairs IR trafficking by trapping it in endosomes, leading to impaired insulin signaling (Zhao et al., 2017). The APOE gene ε4 allele is so far considered the strongest genetic risk factor for AD. These findings are relevant to explain the correlation between T2DM and LOAD (Peila et al., 2002). In addition, this implies that the presence of APOE4 allele and T2DM could act synergistically in AD pathogenesis. Accordingly, the highest risk for AD and the most severe neuropathology is found in individuals with both diabetes and the APOE4 mutation (Peila et al., 2002).

## Obesogenic Diet as a Risk Factor for Cognitive Impairment

Previous studies reported that obesity is associated to memory impairment through insulin resistance in both, young people (Cheke et al., 2016) and cognitively normal older people although the underlying mechanisms remain unclear (Hargrave et al., 2016; Kivipelto et al., 2018). Likewise, in the older people an association between obesity and brain atrophy has been described (Raji et al., 2010). It is widely accepted that obesity favors the emergence of metabolic syndrome affecting peripheral tissues such as liver, pancreas and adipocytes (Cardoso et al., 2017; Kang et al., 2017; Kothari et al., 2017). We have already discussed the observations based on the experimental use of STZ to induce diabetes in rats (de la Monte et al., 2006; de la Monte, 2017). This toxic compound can trigger Type 1 diabetes mellitus by killing insulin-producing cells in the pancreas and, at lower doses, can lead to T2DM and related alterations (Correia et al., 2011; Salkovic-Petrisic et al., 2013; Tong et al., 2017). Indeed, lower STZ doses have been involved in neuronal loss, neuroinflammation, oxidative stress and accumulations of phospho-TAU and Aβ in cortical-limbic structures that characteristically degenerate in AD, leading to impaired spatial learning and memory (Correia et al., 2011). Since STZ is a nitrosamine, a chemical compound that can be found in many foods and other consumables, the question is: could diet be directly involved in the exacerbation of cognitive decline in LOAD? Indeed, several studies suggest that environmental exposure to food additives may play a critical role in the pathogenesis of AD (de la Monte et al., 2018, 2019). In light of the unstoppable increase in LOAD prevalence rates and the widespread use of nitrites and nitrates in foods and agricultural products over the past 30–40 years, the impact of exposure to dietary components as nitrosamines should be reviewed in relation to T3D (de la Monte et al., 2018).

Several studies suggest that there is a link between calorie intake from diets rich in saturated fats or high fat diets (HFD) and the resulting obesity and cognitive deficits (Kohjima et al., 2010; Yarchoan et al., 2014; Pratchayasakul et al., 2015; Moser and Pike, 2017; Sah et al., 2017). By contrast, moderate dietary restriction has been found to improve cognition and, indeed, life expectancy (Parrella et al., 2013). These findings are the reason why studies on the interaction metabolism-AD have gained a great deal of interest. Thus, it is not surprising that HFD has been associated with a large number of metabolic diseases, such as obesity, systemic insulin resistance, metabolic syndrome and T2DM (de la Monte, 2014; Ferreira et al., 2014; Cardoso et al., 2017). Nuzzo et al. (2015) provided evidence that obesity and insulin resistance are involved to inflammation, adipokine dyshomeostasis, oxidative stress and mitochondrial dysfunction, all of them being mechanisms that favor neurodegeneration. In those experiments, mice fed with an HFD showed elevated levels of APP and Aβ40/Aβ42, BACE, GSK3β and TAU proteins, all involved in APP processing and Aβ accumulation. In light of these results, it is clear that the exposure of rodents to a HFD damages their brain in a similar manner to the hallmarks of AD (Pratchayasakul et al., 2015; Ettcheto et al., 2016; Sah et al., 2017). In another study, Bocarsly et al. (2015) also reported negative consequences of HFD in rats. Specifically, HFD has been associated to alterations in brain cortical dendritic spines and a decrease in presynaptic and postsynaptic protein levels, which was related to behavioral cognitive deficits in working memory and cognition (Bocarsly et al., 2015). In turn, Kothari and colleagues reported that HFD may impair brain insulin signaling promoting neuroinflammation, formation of Aβ plaques and neurofibrillary tangles, as well as loss of synaptic plasticity (Sallam et al., 2015). In the same line, Kohjima et al. (2010) also reported that diet-induced insulin resistance is associated with reduced neuronal insulin receptor signaling, leading to an increase in Aβ levels and cognitive loss in the brain of Tg2576 mice. Likewise, an hypercaloric diet increases brain Aβ levels and cognitive alterations in APPswe/PS1dE9 (APP/PS1) mice (Petrov et al., 2015). Similarly, the same murine model under a HFD at an early pre-symptomatic disease stage (3 months old) showed an increase in Aβ1–42 peptide, a decrease in Protein Kinase A (PKA) levels and alterations in the c-AMP Response Element Binding (CREB)/N-methyl-D-aspartate receptor 2B (NMDAR2B)/Peroxisome proliferator-activated receptor gamma coactivator 1-alpha (PGC-1α) pathway (Sheng et al., 2012; Ettcheto et al., 2016; Katsouri et al., 2016; Wang et al., 2017). This mechanism involves the attenuation of the forkhead-like transcription factor 1 (FoxO3a) expression.

There can be found some other several preclinical studies in which the exposure to HFD is associated with a decline in cognitive function. In many of these studies, HFD-induced alterations in peripheral insulin sensitivity lead to a central insulin resistance and biochemical changes related to increased Aβ deposition and neurofibrillary tangle formation (Kothari et al., 2017). For instance, Chua et al. (2012) demonstrated that a reduction in insulin signaling usually precedes the accumulation of Aβ peptide in APP/PS1 mice. In another study developed by our research group, long-term exposure to HFD favored the appearance of Aβ depositions in the brain of C57BL/6J mice (Busquets et al., 2017). This is an intriguing observation because, since these wild-type mice do not develop cognitive loss *per se*, our results implied that HFD maintained for a long time could be enough to damage brain. Moreover, HFD caused alterations in different cell processes, such as normal autophagy and apoptosis, and also enhanced an inflammatory reaction that leads to a decrease in the neuronal precursor cells (Busquets et al., 2017).

Taken altogether, the above-mentioned results reinforce the hypothesis of a metabolic etiology of AD in its sporadic and late onset form. They also confirm that HFD favors Aβ depositions in the brain, a key feature of this disease. In this point, an important question arises: what is the molecular link between diet, T2DM and cognitive impairment? In this regard, Osborne et al. (2016) reported that intrahippocampal infusion of an Aβ33–42 gamma antibody reversed cognitive impairment in rats with HFD-related cognitive loss. Hence, these results stressed the role of soluble Aβ in obesity-mediated cognitive loss and they are in agreement with previous studies hypothesizing that diffusible Aβ oligomers are responsible for neural dysfunction leading to AD (Walsh et al., 2005; Tarasoff-Conway et al., 2015; Xia et al., 2016; Bu et al., 2018; Hurtado-Puerto et al., 2018). Accordingly, Aβ could be a possible connection between the metabolic and the classical amyloid hypotheses of AD, since it binds to the IR and may trigger its internalization at the post-synaptic level, thereby blocking glutamatergic neurotransmission (De Felice et al., 2014; Ribe and Lovestone, 2016; Ahmed et al., 2017). In addition, Aβ oligomers have been found to inactivate IRs *via* the JNKs pathway through a mechanisms that is comparable to the established peripheral effect of JNKs on IRs in T2DM (Zhao et al., 2008; Ma et al., 2009; Freiherr et al., 2013; Lyra E Silva et al., 2019).

## Which Came First: Obesity Or Aβ? The “Chicken or the Egg” Causality Dilemma in Load

Among the plethora of functions performed by the CNS, it also plays a key role in the glucose homeostasis. Indeed, different glucose sensors signals are integrated and processed by the CNS, involved in regulating glucose production, pancreatic hormonal secretion and glucose uptake, maintaining the balanced glucose levels against changing conditions (Cai, 2013; Zheng et al., 2018). The hypothalamus controls several regulatory mechanisms of peripheral glucose homeostasis through the control of various signals from organs and tissues involved in digestion, absorption and storage of nutrients. Neurons responsible for the CNS metabolic balance are found in a sub-region of the ventromedial hypothalamus, called the arcuate nucleus (aRC). They express anabolic peptides such as the neuropeptide Y and agouti-related peptide (aGRP), as well as proopiomelanocortin, which is the precursor of numerous biologically active peptides, including the α-melanocyte stimulating hormone (αMSH) which favors catabolism (Obici et al., 2002; Obici, 2009; Sandoval et al., 2009). This complex machinery includes hormones like insulin, adipokines as leptin, molecules like ghrelin or gut peptides as Glucagon-like peptide-1 (GLP-1) and glucose-dependent insulinotropic polypeptide (GIP; De Felice et al., 2014; Fasshauer and Blüher, 2015). Insulin decreases blood glucose concentrations by suppressing glucose production and upregulating its absorption by peripheral tissues (such as skeletal muscles and fat). Leptin exerts anorexigenic effect when it is released from fat tissue, whereas GLP1 and GIP are secreted from the pancreas during feeding inducing the increase glucose-dependent insulin secretion. However, the CNS also produces some of these molecules and receptors, which can be found in many of its areas including the aRC. Surprisingly, in addition to be capable of trespassing the BBB, insulin can also be secreted autonomously by the brain (Warren et al., 2012; Folch et al., 2013; Nuzzo et al., 2015).

**Figure 1:**
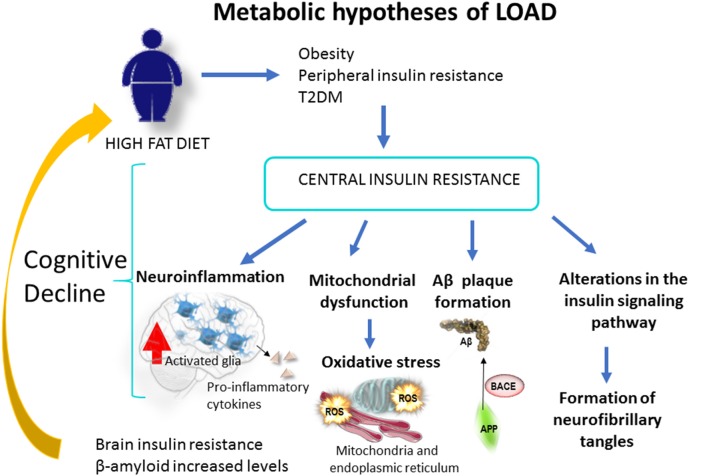
Obesity, caused by a sedentary life or a high fat diet, increases the risk of type 2 Diabetes Mellitus [T2DM; Metabolic hypothesis of Alzheimer’s disease (AD)]. This condition increases the risk of cognitive loss and AD through mitochondrial alteration, neuroinflammation, Aβ production and brain insulin resistance. At the brain level Aβ oligomers trigger peripheral systemic glucose intolerance and insulin resistance in rodents, through a process of hypothalamic neuroinflammation (Clarke et al., 2015, 2018; Lourenco et al., 2019). Likewise, cerebral Aβ could traverse the blood-brain barrier (BBB) and affect peripheral tissues, leading to a peripheral insulin resistance. This mechanism would behave in a positive feedback loop that would worsen over time.

In light of the above-mentioned, it is crucial to understand the potential mechanisms linking AD to obesity and T2DM. Among the hypotheses suggested, there is mounting data supporting an early involvement of Aβ-mediated alterations in hypothalamus leading to peripheral metabolic dysregulation. This may occur even before the appearance of cognitive loss symptoms. In this line of thought, Arrieta-Cruz and colleagues reported that direct administration of Aβ25–35 in the hypothalamus disrupts the regulation of glucose oxidation (Arrieta-Cruz et al., 2015; Arrieta-Cruz and Gutiérrez-Juárez, 2016). In turn, Clarke and colleagues also reported that intracerebroventricular injections of Aβ oligomers trigger peripheral systemic glucose intolerance and insulin resistance in rodents, through a process related with hypothalamic inflammation (Clarke et al., 2015, 2018; Lourenco et al., 2019). Hence, these data reinforce the notion that Aβ affects hypothalamic function, altering peripheral metabolic control and whole-body homeostasis (Obici et al., 2002; Sandoval et al., 2009; Figure 1). Likewise, cerebral Aβ could traverse the BBB and affect peripheral tissues, leading to peripheral insulin resistance. In accordance, it has been demonstrated that plasma Aβ induces insulin resistance in hepatocytes by activating Janus Kinase 2 (JAK2)/STAT3/Suppressor of Cytokine Signaling-1 (SOCS-1) signaling pathway in APP/PS1 mice, suggesting an important role of peripheral Aβ in the regulation of glucose metabolism (Zhang et al., 2013). Moreover, Aβ accumulation also occurs in the pancreas and skeletal muscle which may induce alterations on peripheral glucose metabolism (Roher et al., 2009; Miklossy et al., 2010; Kulas et al., 2017). Likewise, peripheral tissues such as heart, liver, testis, aorta, lung, intestines, skin, as well as the adrenal, salivary, and thyroid glands also produced Aβ peptide (Wang et al., 2017; Wijesekara et al., 2017). Although the implications of peripheral Aβ are still unknown, a contribution of a dynamic maintenance of Aβ levels throughout the body should not be discarded (Selkoe and Hardy, 2016; Bu et al., 2018). Indeed, platelets are a peripheral source of APP and they are able to generate Aβ in a similar manner than neurons, skin fibroblasts, skeletal muscles and cerebrovascular smooth muscle cells (Roher et al., 2009). Hence, peripheral alterations in APP metabolism might constitute a systemic and also a CNS problem in LOAD, hypothesizing that these peripheral Aβ could also contribute to T2DM pathophysiology (Kuo et al., 2000; Shinohara and Sato, 2017). In any case, some recent studies have provided data on the complex mechanistic interactions between T2DM and AD. For instance, Plucińska et al. (2016) demonstrated, using a neuron-specific human BACE1 knock-in mouse model (PLB4) that increased neuronal BACE1 is sufficient to alter systemic glucose metabolism. Therefore, this study also confirms that brain Aβ leads to a peripheral T2DM process. In turn, Sallam and colleagues developed an adipocyte-specific ecto-nucleotide pyrophosphate phosphodiesterase over-expressing transgenic (AtENPP1-Tg) as metabolic syndrome and systemic insulin resistance animal model (Sallam et al., 2015). These mice showed changes in lipid composition of hippocampal synaptosomes, impaired basal synaptic transmission as well as down-regulation of IR expression. The authors concluded that hippocampal molecular and functional integrity become affected by the IR and lipid composition, describing a potential mechanism responsible for the cognitive impairments associated with metabolic syndrome (FFA) and T2DM (Sallam et al., 2015).

Therefore, the process of cognitive impairment could start in peripheral tissues or, at least, could be exacerbated by potential interactions between peripheral tissues and brain. Excessive caloric intake leads to hypertrophy or hyperplasia of adipocytes, resulting in adipose tissue expansion. As a consequence, there is an increased secretion of adipokines, including a broad range of cytokines and chemokines, triggering the recruitment of inflammatory cells to the tissue and a second wave of inflammatory mediators release (Fasshauer and Blüher, 2015). Among these mediators, TNFα and some angiogenic factors could inhibit peripheral insulin signaling. Additionally, other proinflammatory cytokines such as interleukin IL1b and IL6, are able to cross the BBB and exacerbate brain inflammation together with proinflammatory factors produced by microglial cells (Warren et al., 2012; Fasshauer and Blüher, 2015). In turn, high blood insulin concentrations trigger an excessive release of FFA from adipocytes to liver and muscles, leading to exacerbated inflammatory responses and increased accumulation of Aβ. By contrast, hyperinsulinemia decreases insulin transport into the brain. Thus, adipose tissue extension in obesity and T2DM could trigger cognitive loss, reflecting the relationship between the adipose tissue and the brain, thus impacting on its function (Fasshauer and Blüher, 2015). Conversely, hypothalamic soluble Aβ and Tau phosphorylation may contribute to the impairment of the control of peripheral glucose metabolism in patients with AD (Cai, 2013; Chen and Zhong, 2013). This could be mediated by a direct effect of Aβ on central IR and by a mechanism mediated by Tau hyperphosphorylation, which increases intraneuronal insoluble insulin aggregates and downregulates IRs, leading to insulin resistance (El Khoury et al., 2014; Marciniak et al., 2017; Rodriguez-Rodriguez et al., 2017; Gonçalves et al., 2019).

Finally, a study performed by Banks et al. (2018) introduced an additional mechanism whereby metabolic syndrome contributes to cognitive impairment. The authors reported that triglycerides cross the BBB leading to brain leptin and insulin receptors resistance, which has a negative effect on cognition (Banks et al., 2018). These results confirm that modulation of peripheral metabolism, for example lowering elevated levels of triglycerides in the blood, could be a strategy to treat obesity and cognitive impairment associated with CNS resistance to leptin and insulin. In another interesting study, Moreno-Gonzalez et al. (2017) demonstrated that IAPP (amylin) aggregates are able to enhance the aggregation of Aβ, providing a potential additional link between AD and T2DM. Clearly, future studies will provide further keys to understand the relation between cognitive loss, obesity and T2DM (Vazquez-Valls et al., 2011; Mukherjee et al., 2017).

## Role of JNK1 as a Target for Diabetes Type 2 and Obesity

Obesity triggers inflammatory processes that spread through the human body affecting multiple organs and tissues. Consequently, brains of HFD-exposed mice showed neuroinflammation and glial responses (Busquets et al., 2017). In a very recent review on this topic, authors described how HFD, western diet or sugars cause obesity-derived neuroinflammation, affecting brain structures such as the hippocampus, hypothalamus, cortex, brainstem, or amygdala (Guillemot-Legris et al., 2016). Similarly, AD patients exhibit significantly higher concentrations of IL-6 and TNFα in peripheral blood (Swardfager et al., 2010; Zheng et al., 2018).

Regarding the possibility that peripheral chronic inflammation can contribute to cognitive decline and cause LOAD, data show that IR tyrosine kinases trigger the activation of the RAS/MAPKs pathway. This superfamily includes extracellular signal-regulated kinases 1 and 2 (ERK1 and ERK2), p38 and c-JNKs (Coffey, 2014). Moreover, it has been reported that ERK1 and ERK2 play a very significant role in the control of synapses in the learning and memory processes, while the contribution of p38 in associative learning has been described marginally (Sherrin et al., 2010, 2011). Likewise, CREB is the main target of ERK and has a pivotal role in long-term memory and synaptic plasticity in the hippocampus (Suzuki et al., 2011; Teich et al., 2015). Regarding to JNK family, it consists of three members, JNK1 (Mapk8), JNK2 (Mapk9) and JNK3 (Mapk10; Sabio and Davis, 2010; Auladell et al., 2017; Solinas and Becattini, 2017). It has been proposed that the JNKs are involved in memory formation during learning under stressful conditions through the regulation of their activity (Coffey, 2014). Short-term activation of JNKs will temporarily boost memory performance, whereas prolonged activation of JNKs may be a contributing factor to memory deficit and even neurodegeneration (Sherrin et al., 2011). The pro-inflammatory cytokine TNF-α activates JNK1 (Sabio and Davis, 2010; Solinas and Becattini, 2017). Indeed, it has been shown that JNKs are activated in obese humans, thus, JNK1 could be implicated in the mechanism of obesity-induced insulin resistance (Belgardt et al., 2010). Therefore, the characterization of JNK isoforms in the hippocampus and their role in memory processes seems paramount to understand the links among obesity, T2DM and LOAD (Solinas and Becattini, 2017).

**Figure 2:**
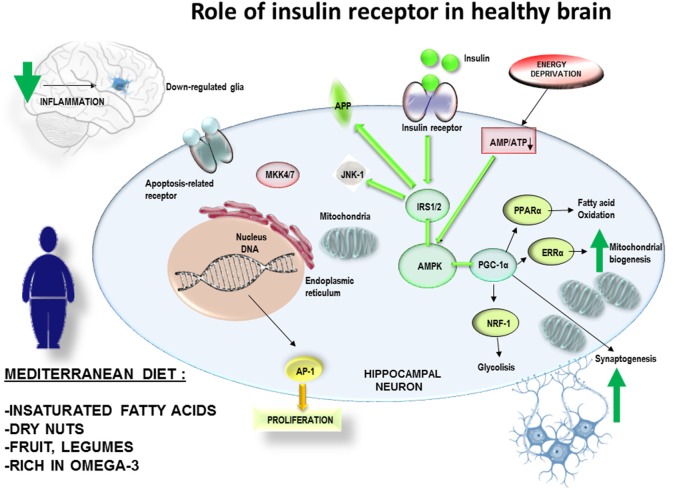
A suitable diet with natural products, for example rich in omega 3, antioxidants et cetera. can be a non-pharmacological complement for the prevention of obesity. Therefore, it would also have effects on the decrease of peripheral and central inflammatory processes. This mechanism would have effects on the prevention of central insulin resistance and improvement of the cognitive processes. The adherence over time to MedDiet would reduce the inflammatory levels in brain, with a decrease in the generation of mitochondrial stress andJUN N-terminal Kinases-1 (JNK-1) activation. It would allow insulin receptor pathway to fine tune the mitochondrial biogenesis according to the ATP/ADP intracellular balance of neuronal cells related to synaptogenesis and neuronal plasticity.

In the hippocampus, JNKs have both presynaptic and postsynaptic localizations and can regulate proteins from synaptic vesicles, such as synaptotagmin-4 (Sherrin et al., 2010; Nisticò et al., 2015). JNKs also regulate second messenger systems such as cytosolic phospholipase A, cytoskeletal elements (i.e., MAP2, TAU), nuclear hormone receptors (such as the glucocorticoid receptor) or transcription factors, including c-Jun (a member of the activator protein-1, AP-1), activator transcription factor (ATF)-2, CREB (calcium/cAMP) and Elk-1 (Sabio and Davis, 2010; Kant et al., 2013). Hence, all these substrates are potential JNKs targets during the learning process. Moreover, as previously mentioned, JNKs may be central mediators of many of the deleterious consequences of obesity, such as impaired glucose metabolism and insulin resistance (Belgardt et al., 2010). This hypothesis was proposed after exposing JNK1 knockout mice to an HFD. Surprisingly, these animals were protected from the development of impaired glucose tolerance and insulin resistance (Pal et al., 2016). Indeed, activated JNK1 phosphorylates the insulin receptor substrate 1 (IRS-1) in the serine residues (IRS-1pSer), blocking the insulin pathway and causing a peripheral resistance to this hormone (Solinas and Becattini, 2017). Phosphorylation in S307 is a mechanism by which the activation of JNK can directly antagonize insulin action. Therefore, phosphorylation of IRS1 following the activation of JNK1 has a key role in the insulin resistance mechanism and obesity process in mammals. Likewise, the diabetic status alters the signaling pathway downstream of IR. Among others, it is relevant the energy alteration sensing pathway comprising the AMP-activated protein kinase (AMPK)/sirtuin (SIRT)/peroxisome proliferator-activated receptor-γ coactivator α (PGC-1α; Fernyhough, 2015). In fact, the intracellular ATP/ADP balance is regulated by AMPK, which acts as a master sensor that, in turns, also control the glucose and fatty acids consumption, and the mitochondrial biogenesis through PGC-1α activity. The energy balance inside the hippocampal neurons allows for the formation, maintenance and reorganization of synapses, all of them critical processes for brain development and appropriate responses generation from neuronal circuits to environmental challenges (Cheng et al., 2012). These authors demonstrated how PGC-1α activity increases dendritic spines and enhances the molecular differentiation of synapses in cultured hippocampal cells (Figure 2). Then, in light of evidences, it could be hypothesized PGC-1α as a molecular link between metabolic alterations involving brain diabetic status and cognitive impairment.

In addition, other kinases such as nuclear kappa-β kinase inhibitor (IKK) play a crucial role in the development of insulin resistance associated with obesity, in both peripheral and CNS (Sabio and Davis, 2010; Kant et al., 2013; Nisticò et al., 2015). Likewise, recent studies suggest that activated protein RNA kinase, also known as protein kinase R (PKR), plays an important role in insulin resistance induced by a HFD diet (Taga et al., 2018). Based on this activation sequence, JNK1 modulation looks like a promising molecule for future development of novel therapeutic targets aiming to prevent T2DM. Consequently, selective inhibitors like Licochalcone A, may be suitable approaches to treat T2DM-related cognitive loss (Busquets et al., 2018).

Unfortunately, there is a lack of natural or synthetic molecules capable to modulate the activity of JNKs so far. Among the extensive effort in exploring therapeutic interventions focusing on JNK activity, the compound SP600125, a JNK inhibitor, has been the best characterized (Gao et al., 2017). However, the application of SP600125 is very limited due to its low target selectivity, and its clinical efficacy is restricted due to its poor aqueous solubility (Gao et al., 2017). Notwithstanding, selective, water-soluble and brain-penetrant JNK inhibitors have been recently tested (Kumar et al., 2016). I.P. and i.c.v. administrations of SR11935, SR3306 and JNK2/3 isoform-selective inhibitors indicated possible anorectic effects (Ryu et al., 2016).

## The Complex Treatment of Late Onset Alzheimer’s Disease: More Than Antidiabetic Drugs

At least, a link between cognitive loss and T2DM involves dysregulation of CNS circuits that control hepatic glucose production. Thus, targeting these circuits could be a novel potential strategy for the development of more effective therapies resulting in both, improved glucose regulation and cognitive performance. In this regard, our research group has already discussed the potential cognitive benefits from drugs originally addressed to treat T2DM (Batista et al., 2019; Forny-Germano et al., 2019). Indeed, some molecules used for the treatment of T2DM have shown neuroprotective effects in preclinical models of AD. These drugs included intranasal insulin, sulfonylureas, PPARγ agonists, metformin and GLP-1 receptor agonists such as exendin-4, liraglutide and lixisenatide (de la Monte, 2017; Cummings et al., 2018; Batista et al., 2019). This opens a promising perspective for these antidiabetic drugs. For instance, new formulations of dual GLP-1/GIP, and the triple combination GLP-1/GCG/GIP agonists, which are the most effective drugs for weight loss, have been evaluated to treat LOAD (Camins et al., 2019). Likewise, the pharmacological combination of GLP-1/GCG/GIP has been shown to prevent the decline of brain glucose metabolism in animal models (Capozzi et al., 2018). Molecularly, the triagonist upregulated the expression of CREB, reverted cognitive impairment and enhanced Long-term potentiation (LTP) in preclinical models of AD (Tai et al., 2018). The activation of CREB by phosphorylation at Ser133 (S133p-CREB) is a critical step for memory formation and LTP maintenance since the downstream genes are involved in synaptic formation, neuronal plasticity and neurogenesis (Ettcheto et al., 2018a). However, taking into account the multidisciplinary nature of AD, more than one drug should be administered to reach a complete treatment capable of slowing the neurodegenerative process. Therefore, a combination of drugs that acts on different pathways involved in the neuropathology of the disease (i.e., amyloidogenic pathway, metabolism disorders, excitotoxicity or neuroinflammation) could be the optimal choice to treat AD. Furthermore, these drugs should be administered as soon as possible to delay the process of cognitive loss.

The antidiabetic drug pioglitazone has been evaluated for AD treatment in the so-called TOMMORROW clinical trial (ClinicalTrials.gov Identifier: NCT01931566). This phase III study assess delay of onset of MCI-AD in cognitively normal subjects between 65 and 83 years of age. The study has two objectives: the first is a new genetic test to determine whether participants are at risk of developing a mild cognitive impairment related to AD (MCI-AD) in the 5 years of study based on a genetic biomarker composed of TOMM40 and APOE genotypes and age used at the time of study incorporation. The second objective is to evaluate the efficacy of pioglitazone to delay the onset of MCI-AD in cognitively normal people who are at high risk of developing MCI-AD (Roses et al., 2014). The study will include 3,500 subjects. However, Takeda Pharmaceutical Company Limited after a preliminary analysis of the results reported that pioglitazone seems that was not effective in MCI-AD prevention.

The studies evaluating the administration of nasal insulin in the fight against forgetting (SNIFF) consists of a multicenter, double-blind, placebo-controlled phase 2/3 trial sponsored by the Cooperative Study of AD (ClinicalTrials.gov Identifier: NCT01767909). The study aims to evaluate the efficacy of intranasal administered insulin on cognition, entorhinal cortex and hippocampal atrophy, and cerebrospinal fluid (CSF) biomarkers in amnestic mild cognitive impairment (aMCI) or mild AD. Thus, it will study AD biomarker profile, gender, or APOE-ε4 allele carriage predict treatment response. According to the hypothesis after 12 months of treatment with nasal insulin compared to placebo, subjects would improve performance on a global measure of cognition, on a memory composite and on daily function. The results of the study have not yet been published.

Finally, as we have previously stated, it has been reported that Memantine improves the metabolic consequences produced by HFD in the APP/PS1 mice model of familial AD (Ettcheto et al., 2018b). These results demonstrate new possibilities into the role of Memantine not only in the occurrence of AD treatment, but also into its potential application in peripheral metabolic disorders where Aβ could play a role, as is the case of T2DM (Ahmed et al., 2017; Folch et al., 2018).

## Non-pharmacological Strategies to Enhance Cognitive Performance: Diet Interventions

In 2018, the group of Dr. Marta di Carlo published new results in which they discussed the beneficial effects of a natural dietary supplement (NDS) containing *Curcuma longa*, silymarin, guggul, chlorogenic acid and inulin (Nuzzo et al., 2018). They showed that NDS exerts neuroprotective effects in HFD mice by reducing brain fat accumulation, oxidative stress and inflammation, as well as by improving brain insulin resistance (Nuzzo et al., 2018). Hence, it seems that dietary content can enhance or destabilize the delicate machinery that allows neurons to survive, which leads to the following question: could diet influence cognitive performance in human populations?

As it is common in science, the answer goes probably beyond a simple “yes” or “no,” but diet is indeed an important modifiable lifestyle factor related to the development of many pathologies and, among them there is all the different subtypes of dementia (Gustafson et al., 2015). The studies leaded by Dr. Mia Kivipelto were the first to show that beneficial midlife dietary changes are associated with a reduced dementia risk later in life (Sindi et al., 2018). Their results highlighted the importance of targeting dietary patterns, describing how the combination of determined food may have synergistic effects, thus further potentiating their benefits. A meta-analysis by Hill et al. (2018, 2019) also revealed an effect of diet on AD biomarkers. With 2,726 records, the review supported the notion that diet and nutrition display potential for non-pharmacological strategies to improve the prognosis and prevent AD (Hill et al., 2018). More recent investigations also showed the potential cerebral benefits of diet interventions in human populations. For instance, results from the Finnish Geriatric Intervention Study to Prevent Cognitive Impairment and Disability (FINGER), which included 1,260 participants at-risk of cognitive decline (60–77 years), allowed to conclude that, in fact, dietary changes seem to play a key role in preventing cognitive loss (Lehtisalo et al., 2019). In this study, the ingestion of a balanced diet was associated with positive changes in executive function, especially in the intervention group, after a 2-years follow-up. Hence, these new approaches would show effects in the long term and would be effective if they were to be followed for a long-term. Thus, becoming complementary and preventive in the long run.

This leads us to the next question: is there any particular diet to adhere in order to prevent cognitive loss? In this regard, a growing body of evidence associate the Mediterranean diet (MedDiet) to preservation of cognitive performance in human populations. MedDiet is characterized by a high intake of fruits, vegetables, legumes, fish, whole grains, nuts, and olive oil, a moderate consumption of dairy products and wine, and a low intake of red and processed meats and foods that contain high amounts of added sugars (Trichopoulou et al., 2003). Indeed, recent results from non-Mediterranean populations suggest that higher MedDiet adherence is associated with higher global cognitive performance and brain structural integrity, as well as decreased risk of AD and vascular dementia (VaD; Karstens et al., 2019). In this line, the geographic location of our research group allows us to describe our own experience from a closer point of view, studying human populations naturally adhered to MedDiet due to cultural reasons. The PREDIMED (in Spanish: *PREvención con DIeta MEDiterránea*) study is a huge project that has published more than 200 articles in indexed journals during the last decade. The results from primary prevention trials reported that long-term adherence to a MedDiet, supplemented with either extra-virgin olive oil or nuts, reduced cardiovascular disease (Hu et al., 2013). Also, other studies on the antioxidant effects of walnuts proved evidence on their effects (Bulló et al., 2010). The results from the PREDIMED project rapidly spread through other metabolic aspects related to a MedDiet adherence. Of note, Salas-Salvadó et al. (2016) reviewed the role of MedDiet on preventing T2DM and stated that the role of the MedDiet on the prevention and management of T2DM and metabolic syndrome proves true according to the data of the study. In turn, metabolomic studies allowed to identify plasma compounds with potential to predict both insulin resistance and incident T2DM (Papandreou et al., 2019). This is relevant since, both in T2DM and LOAD, prevention could be a key factor, and there is still a lack of clear molecular markers allowing to detect at-risk candidates soon enough to reverse the damaging effects of these diseases.

**Figure 3:**
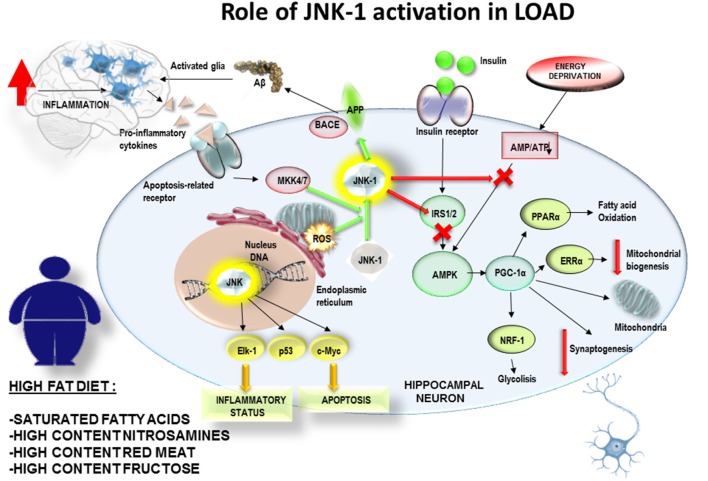
The exposure to high fat diets over time, enriched in saturated fatty acids, would promote the glial activation and an increased mitochondrial oxidative stress. All those stressing factors would activate JNK-1 resulting in an impairment of insulin receptor pathway, causing and imbalance in ATP/ADP levels and a failure to control mitochondrial biogenesis and to maintain the synaptic connexions. This hypothesis would explain how JNK-1 should be a key factor linking diet and insulin signaling to synaptogenesis failure.

Conclusively, a PREDIMED study examined the effect of T2DM on cognitive performance, specifically executive function tasks, in a large cohort of 6,823 patients above 55 years of age (Mallorquí-Bagué et al., 2018). In this cross-sectional study, T2DM (including illness duration), higher Body Mass Index and lower mood were linked to lower cognitive function in older individuals with conditions like overweight/obesity and metabolic syndrome. Of note, MedDiet includes a moderate intake of red wine and extra virgin olive oil, both of them rich in polyphenolic compounds, such as resveratrol, oleuropein, hydroxytyrosol and their derivatives, which have shown anti-inflammatory effects on microglia on *in vitro* and *in vivo* studies (Hornedo-Ortega et al., 2018). According to these observations, it can be hypothesized the adherence over time to MedDiet would reduce the inflammatory levels in brain and the generation of mitochondrial stress together with JNK-1 activation. It would allow insulin receptor pathway to fine tune the mitochondrial biogenesis according to the ATP/ADP intracellular balance of neuronal cells related to synaptogenesis and neuronal plasticity (Figure 2). By contrast, the exposure to high fat diets, enriched in saturated fatty acids, would promote the glial activation and mitochondrial oxidative stress. All those stressing factors would activate JNK-1 resulting in an impairment of insulin receptor pathway, causing and imbalance in ATP/ADP levels and a failure to maintain the synaptic connections (Figure 3). This hypothesis would explain how JNK-1 should be a key factor linking diet and insulin signaling to synaptogenesis failure.

Interestingly, one of the latest reports from the PREDIMED project revealed changes in circulating MicroRNAs (miRNAs). The GLYNDIET study is a 6-month, parallel, randomized clinical trial conducted on overweight and obese subjects. Results from GLYNDIET recently showed that the intake of an energy-restricted low-glycaemic index diet down-regulates circulating miRNA-361 more than an energy-restricted high-glycaemic index, regardless of the magnitude of the weight loss Giardina et al., 2019). Furthermore, Dr. Inestrosa stated in a seminal review that miRNAs may be a molecular link in the complex relationship between metabolic syndrome and AD (Codocedo et al., 2016) and, a recent systematic review extracted all miRNAs found to be significantly deregulated in peripheral blood and cross-referenced them against the miRNAs deregulated in the brain at Braak Stage III (Swarbrick et al., 2019). This resulted in a group of 10 miRNAs; hsa-mir-107, hsa-mir-26b, hsa-mir-30e, hsa-mir-34a, hsa-mir-485, hsa-mir200c, hsa-mir-210, hsa-mir-146a, hsa-mir-34c, and hsa-mir-125b that could be potentially involved in the regulation of these mechanisms. The authors hypothesized that these molecular markers could be deregulated early in AD, nearly 20 years before the emergence of clinical symptoms (Swarbrick et al., 2019). Yet, PREDIMED results on dysregulated circulating miRNAs in obesity and T2DM do not match with those from AD patients. Despite these negative preliminary observations, undoubtedly further studies on circulating miRNAs could open a new therapeutic perspective for patients affected by LOAD.

## New Perspectives in the Near Future

As it has been discussed in this review article, recent results from multiple studies have contributed to reinforce the proposed concept of T3D. In addition, from all the gathered data, it is clear that targeting LOAD early stages, before widespread neurodegeneration has occurred, is likely to produce the best clinical outcome. However, detection of individuals at this stage is still difficult. Consequently, new and reinforced efforts should be made towards the discovery and description of biomarkers that will allow for the early detection of pre-clinical candidates for T2DM and/or LOAD. Clearly, a huge effort will be necessary to overcome this molecular complexity just like in the new formulation of new and more effective treatments for LOAD. For now, it seems that the testing of more anti-T2DM drugs with beneficial effects against cognitive impairment has a certain promising future.

## Author Contributions

All the co-authors of this research (JF, JO, ME, OB, ES-L, ACano, TE-J, GC, CB-Z, MG, CA, MB and ACamins) have directly participated in the planning, execution of the manuscript. All authors have read and approved the final version submitted.

## Conflict of Interest Statement

The authors declare that the research was conducted in the absence of any commercial or financial relationships that could be construed as a potential conflict of interest.
